# Self-organized spatiotemporal quasi-phase-matching in microresonators

**DOI:** 10.1038/s41467-025-59215-1

**Published:** 2025-05-01

**Authors:** Ji Zhou, Jianqi Hu, Marco Clementi, Ozan Yakar, Edgars Nitiss, Anton Stroganov, Camille-Sophie Brès

**Affiliations:** 1 Photonic Systems Laboratory (PHOSL), STI-IEM, École Polytechnique Fédérale de Lausanne, CH-1015, Lausanne, Switzerland; 2 LIGENTEC SA, EPFL Innovation Park, CH-1024, Ecublens, Switzerland; 3 Present Address: Department of Electrical and Electronic Engineering, The University of Hong Kong, Hong Kong, China; 4 Present Address: Dipartimento di Fisica “A. Volta”, Università di Pavia, Via A. Bassi 6, 27100, Pavia, Italy

**Keywords:** Nonlinear optics, Nanophotonics and plasmonics

## Abstract

Quasi-phase-matching (QPM) is a widely adopted technique for mitigating stringent momentum conservation in nonlinear optical processes such as second-harmonic generation (SHG). It effectively compensates for the phase velocity mismatch between optical harmonics by introducing a periodic spatial modulation to the nonlinear optical medium. Such a mechanism has been further generalized to the spatiotemporal domain, where a non-stationary spatial QPM can induce a frequency shift of the generated light. Here we demonstrate how a spatiotemporal QPM grating, consisting in a concurrent spatial and temporal modulation of the nonlinear response, naturally emerges through all-optical poling in silicon nitride microresonators. Mediated by the coherent photogalvanic effect, a traveling space-charge grating is self-organized, affecting momentum and energy conservation, resulting in a quasi-phase-matched and Doppler-shifted second harmonic. Our observation of the photoinduced spatiotemporal QPM expands the scope of phase matching conditions in nonlinear photonics.

## Abstract (web summary)

Quasi-phase-matching (QPM) is crucial in nonlinear optical processes by compensating for phase velocity mismatch. Here, the authors demonstrate a spatiotemporal QPM grating by all-optical poling in silicon nitride microresonators expanding the scope of phase-matching in conditions in nonlinear photonics.

## Introduction

Efficient optical frequency conversion via nonlinear light-matter interaction generally requires both energy and momentum conservation among participating photons. Momentum conservation (i.e., phase velocity matching) is, however, often hindered by the chromatic dispersion of nonlinear optical media^1^. An effective strategy to overcome this limitation is quasi-phase-matching (QPM), initially proposed by Armstrong and coworkers in 1962^2^, which introduces an ordered spatial modulation of the nonlinear susceptibility to compensate the momentum mismatch between optical waves. To date, QPM is a widely used technique to enable second-order (*χ*^(2)^) nonlinear processes like second-harmonic generation (SHG), sum/difference-frequency generation, and spontaneous parametric down conversion. Highly efficient SHG has been achieved by periodic poling of non-centrosymmetric materials such as lithium niobate (LN) and KTiOPO_4_ (KTP) bulk crystals^3–5^, as well as integrated thin-film LN waveguides and microresonators^6,7^. In recent years, following the work carried in doped silica fibers^8–11^, there has also been a growing interest in realizing such *χ*^(2)^ functionalities in integrated centrosymmetric media, such as silicon^12,13^, and silicon nitride (Si_3_N_4_)^14–22^. Despite lacking an intrinsic second-order nonlinearity, these materials can be endowed with an effective *χ*^(2)^ by breaking the inversion symmetry through the application of electric or optical fields.

Amidst these, all-optical poling (AOP) has been an effective means to induce *χ*^(2)^ nonlinearity. Requiring only moderate power, it allows for the optical inscription of *χ*^(2)^ and self-configuration of QPM. This process is enabled by the coherent photogalvanic effect (CPE), wherein the interference among multi-photon absorption processes results in the emergence of coherent currents, and yields the inscription of a static electric field inherently supporting the required QPM condition^23,24^. In Si_3_N_4_ microresonators, AOP has allowed for SHG with high conversion efficiency (CE)^19^ and broad reconfigurability^20^.

**Fig. 1:**
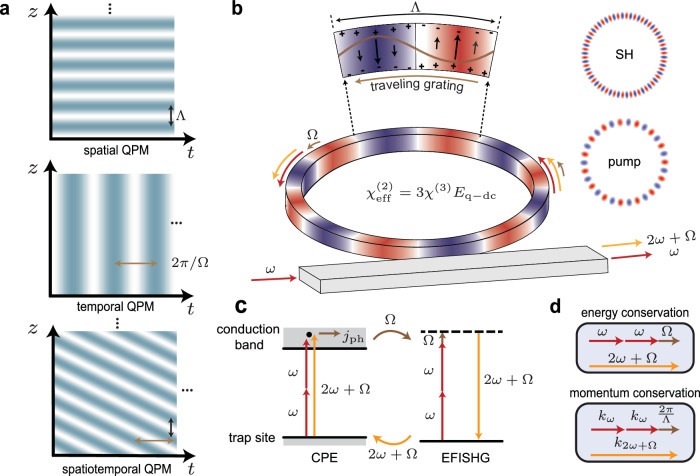
Self-organized spatiotemporal quasi-phase-matching in Si_3_N_4_ microresonators. **a** Various quasi-phase-matching (QPM) schemes. Top: spatial QPM with a period of *Λ* can compensate for the wavevector mismatch of 2*π*/*Λ*, e.g., in a standard second-harmonic generation (SHG) process. Middle: temporal modulation with a period of 2*π*/*Ω* can shift the optical frequency by *Ω*, e.g., in an electro-optic modulation process. Bottom: Spatiotemporal QPM combining both spatial and temporal modulation can simultaneously mitigate momentum and energy mismatch, as demonstrated in processes such as high-harmonic generation and photoinduced SHG within this study. **b** Spatiotemporal QPM based on a self-organized traveling *χ*^(2)^ grating for SHG in a Si_3_N_4_ microresonator. When the doubly resonant condition is met, the pump at frequency *ω* inscribes a *χ*^(2)^ grating traveling at the frequency of *Ω* and leads to the generation of the second-harmonic (SH) at the frequency of 2*ω* + *Ω*. The upper zoom-in shows a period of transverse quasi-DC electric field mediated by separated alternating space-charges, inducing a traveling *χ*^(2)^ grating by ${\chi }_{{{{\rm{eff}}}}}^{(2)}=3{\chi }^{(3)}{E}_{{{{\rm{q-dc}}}}}$. The right insets show the artistic illustration of optical mode profiles for pump and its SH fields. **c** Photoinduced SHG from the interplay between the coherent photogalvanic effect (CPE) and the electric-field-induced SHG (EFISHG) effect. In the CPE, the interference between two pump photons and one SH photon absorption process generates an anisotropic coherent current *j*_ph_, which allows for the inscription of a quasi-dc field *E*_q-dc_. The *E*_q-dc_ enables the quasi-phase-matched EFISHG process for efficient generation of the SH field, which in turn enhances the CPE, forming a self-sustaining positive feedback loop. **d** Momentum and energy conservation diagrams. The spatial and temporal modulation of the traveling *χ*^(2)^ grating affects the momentum and energy conservation, respectively.

While the spatial properties of photoinduced QPM have been extensively investigated^17,18,20^, the dynamics of the process have not been explored, despite a recent work bringing forward the hypothesis of non-stationary solutions by considering photoinduced SHG as optical parametric oscillations^25^. Indeed the nature of the AOP process could entail an additional temporal behavior, similar to photon-phonon interactions (e.g., stimulated Brillouin scattering) and spatiotemporal QPM, where the latter was proposed and demonstrated in high-harmonic generation^26–28^. In this context, the momentum mismatch compensation through the spatial modulation is complemented by a temporal modulation that analogously compensates for an energy mismatch (Fig. 1a).

In this work, we investigate the spatiotemporal dynamics of AOP in Si_3_N_4_ microresonators. Mediated by the CPE, the photoinduced electric field in turn enhances SHG, also known as the electric-field-induced SHG (EFISHG) effect. Remarkably, we find that the dynamics of resonant AOP yields a temporal modulation of the photoinduced nonlinearity, associated with the spatial one, which altogether can be regarded as a nonlinear *χ*^(2)^ grating that travels indefinitely alongside the microring circumference. We investigate the spatial properties of such spatiotemporal QPM by two-photon microscopy (TPM) imaging, while its temporal structures is characterized by self-homodyne and self-heterodyne measurements with a frequency-doubled reference. From the latter, we observe an additional frequency (energy) offset from the typical SHG process, which can be interpreted as a Doppler shift from the interaction with a traveling *χ*^(2)^ grating. Our findings shed light on the physics of resonant AOP, providing new concrete experimental evidence for the spatiotemporal QPM^27^ and establishing a comprehensive model for photoinduced nonlinear processes in resonant systems.

## Results

### Principle of self-organized spatiotemporal QPM in microresonators

We begin by describing the photoinduced SHG in a Si_3_N_4_ microresonator (Fig. 1b). When both the pump and its second-harmonic (SH) are doubly resonant, the initial weak light within the SH resonance seeds the CPE, and the interference between single- and two-photon absorption processes generates an anisotropic coherent current^8,9,24^:

1 $${j}_{{{{\rm{ph}}}}}=\beta {({E}_{{{{\rm{pump}}}}}^{*})}^{2}{E}_{{{{\rm{SH}}}}}{e}^{\,i\Delta kR\phi }{e}^{-i{\psi }_{{{{\rm{ph}}}}}}+c.c.$$

where *β* and *ψ*_ph_ are the photogalvanic coefficient and the interaction phase, respectively. *E*_pump_ and *E*_SH_ are the optical fields of the pump and its SH, with *Δk* = *k*_SH_ − 2*k*_pump_ the wavevector mismatch between them. *ϕ* denotes the azimuthal angle along the circumference of the microresonator with a radius of *R*. Both * and c.c. stand for complex conjugate.

The separation of charges via coherent current leads to the creation of a photoinduced electric field, while the concurrent drift current results in its decay. In the steady state, the photoinduced field can be expressed as *E* = − *j*_ph_/*σ*(*I*_pump_, *I*_SH_), with *σ* the conductivity which depends on the pump and SH light intensity *I*_pump_ and *I*_SH_, respectively. Notably, the wavevector mismatch between the inscribing optical fields renders a spatial modulation of the photoinduced electric field with a period Λ = 2π/Δ*k*. Meanwhile, the electric field also imparts an effective *χ*^(2)^ nonlinearity, i.e., ${\chi }_{{{{\rm{eff}}}}}^{(2)}=3{\chi }^{(3)}E$, thus giving rise to EFISHG. The generated SHG further enhances the CPE (Fig. 1c), forming a positive feedback loop that leads to the growth of SH, ultimately limited by the increase of the photoconductivity *σ*. Remarkably, when the SHG process is interrupted, the displaced charges maintain their spatial distribution owing to the insulating nature of Si_3_N_4_ in the absence of excited carriers. The inscription of a long-lasting nonlinear grating is confirmed by electric-field-sensitive etching^11^ and TPM imaging^18,20^.

To gather insights into the AOP dynamics in microresonators, we model the temporal dynamics of doubly resonant SHG with coupled-mode equations (CME) (see Eqs. (5) in Methods). The theoretical analysis provides two important results for efficient SHG by AOP (see Supplementary Note I):

- The inscribed *χ*^(2)^ grating is stable only when the generated SH (*ω*_SH_) lays on the blue side of the SH resonance (*ω*_*s*_), i.e., when the detuning of the SH light satisfies:
  2 $${\omega }_{s}-{\omega }_{{{{\rm{SH}}}}}\approx {\delta }_{s}^{{\prime} } \, < \, 0.$$
  where ${\delta }_{s}^{{\prime} }={\omega }_{s}-2{\omega }_{{{{\rm{pump}}}}}$ with *ω*_pump_ the pump frequency. The approximation is valid as *ω*_SH _= 2*ω*_pump_  + Ω with Ω/2*π* in the sub-kHz range, according to both the theoretical estimation and experimental observation.
- The photoinduced grating exhibits a temporal oscillation with an angular frequency:
  3 $$\Omega \approx \frac{{\kappa }_{s}}{2{\delta }_{s}^{{\prime} }\tau },$$
  where *κ*_*s*_ is the linewidth of the SH resonance and *τ* is the intensity-dependent grating lifetime.

These results, together with the spatial periodic modulation, can be interpreted as a traveling *χ*^(2)^ grating, as schematically shown in Fig. 1b. Such simultaneous spatial and temporal modulation of the *χ*^(2)^ nonlinearity now arises from a *quasi*-static electric field in the microresonator:

4 $${E}_{{{{\rm{q}}}}{-}{{{\rm{dc}}}}} \sim {e}^{i\,\frac{2\pi }{\Lambda }R\phi -i\Omega t},$$

where the electric field travels along the ring circumference with a phase velocity *v* = ΩΛ/2*π* on the order of a few millimeters per second. Intuitively, the traveling nature of the grating can be understood as a consequence of its self-organized nature: as the SH field is a source of the photoinduced electric field and vice versa (see Eqs. (5) in Methods and also Supplementary Note I), these two fields can share an arbitrary phase relation with respect to the pump field, which takes the form of the time-dependent phase shift *φ*(*t*) = Ω*t*. In other words, the grating can travel due to the existence of an unconstrained degree of freedom between pump, SH field and photoinduced electric field.

An important consequence of these findings is that the traveling grating influences both momentum and energy matching, respectively related to the spatial and temporal modulation, as illustrated in Fig. 1d. While spatial QPM is well understood^2^, the energy modification related to its temporal counterpart can be regarded as a Doppler shift imparted by the traveling grating to the generated field. More generally, the phenomenology described here can be set in the framework of the generalized spatiotemporal QPM^27,29^, where the photoinduced SHG in a microresonator involves the scattering of the pump in a nonlinear photonic crystal. In our spatiotemporal QPM case (Fig. 1a bottom), during AOP the CPE organizes a traveling nonlinear grating that effectively doubles the frequency of the pump, with an additional frequency translation of *Ω* satisfying energy conservation.

### Temporal characterization of spatiotemporal QPM

**Fig. 2:**
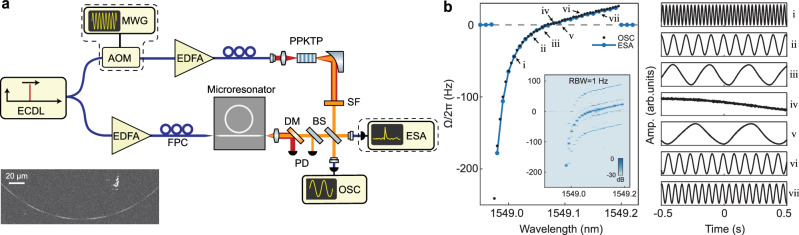
Characterization of the photoinduced second-harmonic frequency offset. **a** Experimental setup for measuring the frequency offset between second-harmonics generated in a Si_3_N_4_ microresonator and a standard frequency doubler. ECDL: external-cavity diode laser; MWG: microwave generator; AOM: acousto-optic modulator; EDFA: erbium-doped fiber amplifier; PPKTP: periodically poled KTiOPO_4_ crystal; SF: spectral filter; FPC: fiber polarization controller; DM: dichroic mirror; PD: photodetector; BS: beam splitter; ESA: electrical spectrum analyzer; OSC: oscilloscope. Both self-homodyne measurements of temporal oscillations using an OSC (excluding dashed boxes) and self-heterodyne measurements of frequency spectra using an ESA are performed. The inset showcases a *χ*^(2)^ nonlinear grating obtained using the two-photon microscopy after the microresonator is poled near the pump resonance at 1549.0 nm. The participating SH mode is TE_30_ inferred from the measured grating period. **b** Left: measured frequency offsets (blue dot-lines: self-heterodyne; black dots: self-homodyne) as functions of the pump wavelength when tuning the laser wavelength across the resonance at 1549.0 nm from blue to red side. The inset shows the spectral map on a logarithmic scale obtained from the self-heterodyne measurement with a 1 Hz resolution bandwidth (RBW) in ESA. Right: temporal oscillation traces measured by the self-homodyne technique for the data points indicated on the left panel (i-vii).

To verify our theoretical predictions, we experimentally investigate the temporal aspect of self-organized QPM. Fig. 2a shows the experimental setup (see “Methods”) used for measuring the frequency offset between the photoinduced SH generated from a Si_3_N_4_ microresonator and a reference SH generated from an external crystal. The Si_3_N_4_ microresonator used in this study has a radius of 158 *μ*m (see “Methods”), and many of its TE_00_ resonances at the telecommunication C-band were found capable to generate SHG via AOP^20^. We perform nonlinear self-heterodyne measurements by recording the optical beating between the two generated SH fields in an electrical spectrum analyzer (ESA) (see “Methods”). This technique offers a 1 Hz resolution (defined by the resolution bandwidth (RBW) of the ESA) in measuring the possible frequency offset, and allows for the unambiguous determination of the exact photoinduced SH frequency. If only purely spatial QPM takes place, the beatnote frequency will be exactly twice of the modulation frequency owing to the SHG process. Otherwise, the presence of the traveling nonlinear grating would impart an additional frequency offset.

The left panel of Fig. 2b presents the steady state frequency offsets experimentally measured at different pump detunings for an AOP instance. In this case, the pump is tuned into the closest resonance near 1549.0 nm from the blue to the red side, and the participating SH mode is TE_30_ mode identified by TPM imaging (Fig. 2a inset). The offset frequencies are retrieved from the ESA spectra (inset). At each detuning, a clear single tone in the range of a few hundred Hz is measured for the frequency offset Ω/2*π*, while the electronic pickup signal is much weaker. Notably, the beatnote signal measured when SH is not generated from the microresonator, is resulted from the beating of the SHG of the weak residual pump and the frequency-shifted local oscillator in the crystal path only. This corresponds to the exact SHG process, serving as a frequency calibration for the offset frequency measurements. In addition, we repeat the experiment in a self-homodyne scheme and record the temporal traces in an oscilloscope (see “Methods”). In this case, we observe temporal oscillations after beating the SH lights, which precisely replicate the trend recorded with the ESA, as shown in Fig. 2b. We note that the frequency offset trace here crosses zero near 1549.08 nm, deviating from the theoretical prediction (Eq. (3)). We attribute this behavior to possibly the perturbation from a neighboring SH mode to the SHG process (see Supplementary Note II).

### Dynamics of photoinduced SHG

**Fig. 3:**
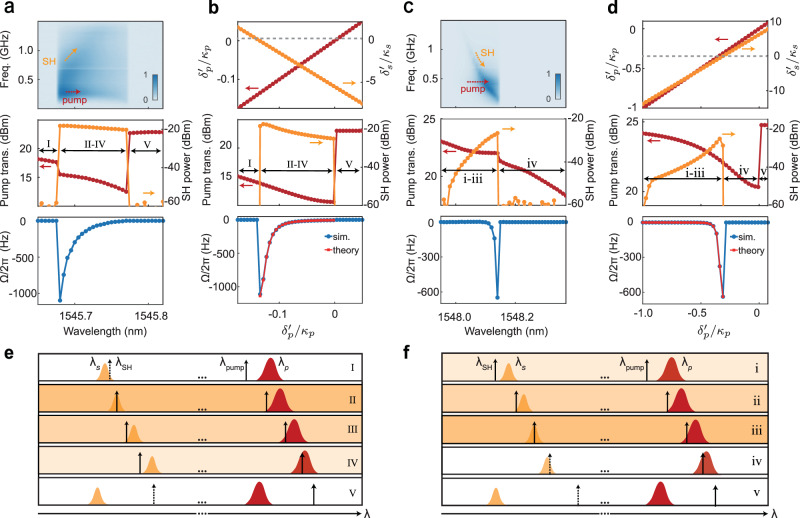
Dynamics of photoinduced second-harmonic generation. **a**, **c** Experimental investigations of photoinduced SHG processes for (**a**) the leading and (**c**) trailing cases. They correspond to the scenarios where the generated SH power decreases and increases with the pump tuning into its resonance, respectively. Top: effective detunings of the pump and SH resonances measured by a vector network analyzer; Middle: Measured pump transmission and generated SH power; Bottom: Measured frequency offset as a function of the pump wavelength. **b,d**, Numerical simulations of photoinduced SHG processes for the leading (**b**) and trailing (**d**) cases. Top: simulated effective detunings of the pump and SH resonances (the gray dashed lines denote ${\delta }_{s}^{{\prime} }=0$); Middle: simulated pump transmission and generated SH power; Bottom: simulated and theoretically predicted frequency offsets as functions of the effective detuning. **e,f**, Schematics of dynamic doubly resonant conditions for the leading (**e**) and trailing (**f**) cases. During pump wavelength tuning from stage I to IV or i to iv, the pump (*λ*_pump_) stays thermally locked to the blue side of its resonance (*λ*_*p*_), while the SH resonance (*λ*_*s*_) varies its relative position from the generated SH wavelength (*λ*_SH_). The leading (trailing) case corresponds to the scenario where the red-shift rate of the SH resonance is two times larger (smaller) than that of the pump resonance when tuning the pump into its resonance. The intensity of the background color indicates the power of the generated SH. At stage V and v, the pump exists the resonance.

To delve deeper into the dynamics of self-organized spatiotemporal QPM in the Si_3_N_4_ microresonator, we perform experiments for several other resonances at C-band that support efficient SHG (see “Methods”). The experimental results can be generally divided into two main cases, depending on the relative red-shift rates of pump and SH resonances while varying the pump wavelength. Two illustrative experimental examples are provided in Fig. 3a, c. In what we call the ‘leading case’ (Fig. 3a), the wavelength at which the maximum absolute value of the frequency offset Ω occurs at the leading edge of the generated SH power trace, and the SH power continuously decreases as the pump is further tuned into the resonance. We use the vector network analyzer (VNA) technique to probe both the pump and SH detunings (see Methods)^20^. The SH resonance is observed to shift away from the generated SH frequency when decreasing the pump frequency (top panel of Fig. 3a). The underlying dynamic doubly-resonant condition is schematically shown in Fig. 3e, where the SH resonance shift rate is regarded as larger than twice the one of the pump resonance (d*λ*_*s*_/d*λ*_pump_ −  2d*λ*_*p*_/d*λ*_pump_ > 0), considering both thermal and Kerr effects. Initially (stage I), the SH resonance is blue-detuned with respect to the doubled frequency of pump, violating the necessary detuning condition (Eq. (2)) and thereby no SH generation is possible. Once the detuning condition is met (stage II), the AOP process is triggered with sufficient pump power, the *χ*^(2)^ grating is inscribed and the generated SH power is suddenly increased. Besides, the VNA response also suddenly appears. Note that, given the relation between the frequency offset and SH detuning (Eq. (3)), this minimal detuning condition not only leads to nearly the maximum generated SH power but also the largest frequency shift. The experimental results are in good agreement with our theoretical prediction. When the pump wavelength is further increased (stages III-IV), the pump detuning reduces slightly but the SH detuning increases significantly (see the VNA response map, where the indicated peak positions approximately correspond to $| {\delta }_{p}^{{\prime} }|$ and $| {\delta }_{s}^{{\prime} }|$^20^), explaining the drop of generated SH power accompanied by the decrease in the frequency offset. In the final stage V, the SHG vanishes after the pump gets out of its resonance.

A different behavior is observed in the ‘trailing case’ (Fig. 3c). In this case, we observe that the SH resonance approaches the generated SH frequency while increasing the pump wavelength (see the VNA response map). This phenomenology can be attributed to the smaller red-shift rate of the SH mode compared to that of the pump (d*λ*_*s*_/d*λ*_pump_  − 2d*λ*_*p*_/d*λ*_pump_ < 0). Different from the ‘leading case’, here the required condition Eq. (2) is promptly satisfied at the beginning of the pump wavelength tuning. The AOP process is therefore triggered as soon as the threshold pump power (see Supplementary Note I) is reached at stage i, even if the detuning conditions for pump and SH are not optimal. When the pump is further tuned closer to the resonance (stages ii–iii), the generated SH power grows significantly, owing to the increased proximity to the doubly-resonant condition (Fig. 3f). The maximum generated SH power is reached in correspondence with the maximum absolute frequency shift. Further, in the early phase of stage iv, the SH power undergoes a sudden drop as ${\delta }_{s}^{{\prime} }\geqslant 0$, therefore the inscribed nonlinear grating is quickly erased preventing the sustained generation of SH.

To corroborate our interpretation, we replicate our experimental findings through numerical integration of our CME model (see Eqs. (5) in Methods and Supplementary Note I). For simplicity, we set the effective detunings at pump and SH to change linearly in the simulation, in qualitative agreement with the behavior retrieved from the experimental VNA maps. In the simulation, we intentionally set the pump power to zero when the pump is out of resonance (${\delta }_{p}^{{\prime} }\geqslant 0$), to replicate the experimental triangular-shaped pump transmission^30^. The changes in detunings result in the corresponding variations in the pump transmission, generated SH power and also the frequency offset. Figure 3b and d show the simulation results corresponding to the ‘leading’ and ‘trailing’ cases, respectively. Numerically we confirm again that the SH can only be generated when the detuning satisfies the condition Eq. (2). A small discrepancy between simulation and experiment is the jumps in the pump transmission, typically observed when the *χ*^(2)^ grating is inscribed (erased). Such jumps may be attributed to the change of the pump detuning due to the sudden presence (disappearance) of strong SH inside the microresonator^31^.

## Discussion

We present in this work the first observation of self-organized spatiotemporal QPM in Si_3_N_4_ microresonators. Our experimental findings confirm the existence of a photoinduced traveling *χ*^(2)^ grating at the steady states of the AOP process. The traveling-wave nature manifests itself as a unidirectional sub-kHz Doppler frequency shift to the generated SH, characterized by both self-homodyne and self-heterodyne measurements. For the quasi-static photoinduced electric field, it is worth stressing that, unlike the optical pump and SH fields, it is not associated with a resonant mode as opposed to the hypothesis initially proposed in ref. ^25^, which is in contradiction with our experimental observations. First, the resonant mode for the experimentally measured frequency offset, if they existed, would entail a resonance wavelength much larger than the chip itself. In addition, the continuous tuning of the frequency shift Ω suggests the absence of resonant modes in the sub-kHz range. Instead, our model relies on the first-order CPE dynamics^23,24^, where the existence of a characteristic wavelength Λ and frequency Ω emerge respectively from the wavevector mismatch and optimal feedback of the system, leading to a non-stationary steady state.

The phenomenon we observe in this work fits well in the framework of spatiotemporal QPM, bearing similarities with the physics of high-harmonic generation from photoinduced gratings^26,32^. Our observation resembles the spatiotemporal modulation ubiquitous in inelastic light scattering processes, such as Brillouin and Raman scattering^33,34^, which however entail the interaction between photons and phonons. Analogies can also be drawn with photoinduced space-charge waves by two-wave mixing in photorefractive crystals^35,36^. In this case, moving Bragg gratings can be created by the interference of two frequency-detuned light beams^37^ or the application of alternating electric field^38^. Finally, a similar phenomenon has been observed in nonlinear angular Doppler experiments, where the SHG in rotating nonlinear crystals was shown to follow both energy and angular momentum conservation conditions^39^.

The developed dynamical model for resonant AOP is of fundamental importance in nonlinear integrated photonics. It provides a theoretical foundation for integrated *χ*^(2)^ frequency converters in amorphous materials^19,20,22,40,41^, unveiling the necessary detuning conditions for efficient SHG as well as explaining the threshold behavior of the process^19,20,25^ (see Supplementary Note I). In this perspective, further optimization can be envisaged by leveraging controls over interaction parameters, such as increasing the out-coupling of the SH mode or with a better regulation of the relative mode detuning, now dependent exclusively on pump thermal tuning. The latter could be improved, for example, with the use of nonlinearly coupled but linearly uncoupled resonators, where the detunings of participating modes could be independently tuned to reach the optimal condition^42–44^. Finally, the developed model can be generalized to *χ*^(2)^ nonlinear processes broadly, such as sum/difference-frequency generation^24,45^, parametric down-conversion^46^, *χ*^(2)^-assisted frequency comb generation^21,47^, comb *f* − 2*f* self-referencing^17^, and self-injection locked frequency doubling^22,48^, among other *χ*^(2)^ functionalities relying on photoinduced nonlinearities.

## Methods

### Theoretical modeling

In the absence of pump depletion, the temporal dynamics of AOP-enabled SHG in a microresonator is governed by (see Supplementary Note I):

5 $$\begin{array}{rcl}&&\frac{\partial {A}_{p}}{\partial t}=-\left(\frac{{\kappa }_{p}}{2}+i{\delta }_{p}^{{\prime} }\right){A}_{p}+\sqrt{{\kappa }_{p}{\eta }_{p}{P}_{{{{\rm{in}}}}}}\\ &&\frac{\partial {A}_{s}}{\partial t}=-\left(\frac{{\kappa }_{s}}{2}+i{\delta }_{s}^{{\prime} }\right){A}_{s}+i\left({\gamma }_{spip}{A}_{i}\right){A}_{p}^{2}\\ &&\frac{\partial {A}_{i}}{\partial t}={\beta }^{{\prime} }{\left({A}_{p}^{*}\right)}^{2}{A}_{s}{e}^{-i{\psi }_{{{{\rm{ph}}}}}}-\frac{{A}_{i}}{\tau }\hfill\end{array}$$

where the subscripts $\left\{p,s,i\right\}$ are used to denote the intracavity optical pump field, intracavity optical SH field and photoinduced electric field, respectively. Here $| {A}_{p(s)}{| }^{2}={\int}_{V}dv{\epsilon }_{p(s)}{\left\vert {E}_{p(s)}\right\vert }^{2}$ and ${\left\vert {A}_{i}\right\vert }^{2}=\frac{1}{2}{\int}_{V}dv{\epsilon }_{i}{\left\vert {E}_{i}\right\vert }^{2}$ represent their modal energy. Note that we include the photoinduced electric field in our coupled mode theory to mathematically describe its temporal dynamics. However, we stress that it is not a resonant mode of the system and it follows instead a first-order CPE dynamics. *κ*_*p*_ and *κ*_*s*_ are the total loss rates (linewidths) of the optical pump and SH fields, ${\delta }_{p}^{{\prime} }={\omega }_{p}-{\omega }_{{{{\rm{pump}}}}}$ and ${\delta }_{s}^{{\prime} }={\omega }_{s}-2{\omega }_{{{{\rm{pump}}}}}$ are effective detunings (i.e., considering the Kerr/thermal effects) of the pump (*ω*_pump_) and its SH (*ω*_SH_) from the respective resonance frequencies *ω*_*p*_ and *ω*_*s*_. *η*_*p*_ is the pump coupling coefficient, *P*_in_ is the pump power in the bus waveguide, *γ*_*spip*_ is the nonlinear coupling parameter for EFISHG effect, ${\beta }^{{\prime} }$ is the effective photogalvanic coefficient incorporating spatial overlap between interacting fields, and *τ*  = *ϵ*_*i*_/*σ* is the lifetime of the photoinduced grating (i.e., electric field) with *ϵ*_*i*_ the static permittivity of the material.

In numerical simulations, the initial condition for initiating SHG involves assigning a small value to the photoinduced electric field. For the temporal evolution of fields, the effective detuning ${\delta }_{s}^{{\prime} }$ are set to change linearly with ${\delta }_{p}^{{\prime} }$. In the steady state, the pump transmission, generated SH power (scaled to the experimental level), and frequency offset are extracted and plotted in Fig. 3b, d.

### Si_3_N_4_ microresonator

The Si_3_N_4_ microresonator employed in this study is identical to the one with 146 GHz FSR (ring radius of 158 *μ*m) used in ref. ^20^. It is fabricated by LIGENTEC using its AN-technology platform. The bus and ring waveguides have the same cross-section of 1.7 × 0.5 *μ*m^2^ and are buried in SiO_2_ cladding, supporting a number of transverse electric (TE) modes from TE_00_ to TE_40_ in SH band. The loaded Q factors are in the level of 0.73 × 10^6^ for TE_00_ resonances at pump wavelengths in C-band.

### Experimental setup

In the experiment, the light from a tunable continuous-wave laser at telecom C-band is firstly divided into two branches. For the self-heterodyne measurement, the upper branch is frequency shifted by an acousto-optic modulator (AOM) at 92 MHz, then amplified and focused onto a periodically poled KTP crystal for frequency doubling. The generated SH from the crystal, after the pump filtering, serves as a SH frequency reference. In the lower branch, the amplified pump is coupled to a Si_3_N_4_ microresonator via a lensed fiber for photoinduced SHG. Here the pump wavelength is slowly scanned until the pump and SH become doubly resonant, triggering the AOP process and generating the SH. The output pump and SH light from the chip are collected using a microscope objective, then separated by a dichroic mirror and measured at respective photodetectors. The measured SH power is not calibrated thereby lower than its actual generated power level. A portion of the generated SH light from the chip and the reference crystal are combined at a beamsplitter, and their optical beating signal is recorded by a ESA at the RBW of 1 Hz. For the self-homodyne measurement, the AOM in the upper branch is bypassed and the temporal beating traces between the SH signals generated from the two branches are recorded by an oscilloscope.

### Measuring VNA response during the AOP process

The effective detunings of pump and its SH (${\delta }_{p}^{{\prime} }$ and ${\delta }_{s}^{{\prime} }$) are tracked by weakly phase modulating the pump with an electro-optic modulator, and measuring the response with a fast photodetector using a VNA (not shown in Fig. 2a). The VNA responses are recorded when varying the pump wavelength, and the detailed working principle of the technique can be found in ref. ^20^. Note that the transmitted pump power (*P*_*p*_), generated SH power (*P*_*s*_), and the frequency offset (Ω/2*π*) are measured when the VNA sweeping is off.

### TPM imaging

We use the TPM technique to image the *χ*^(2)^ grating inscribed in the Si_3_N_4_ microresonator^18,20^. A Ti:sapphire laser with horizontal polarization is focused on the *χ*^(2)^ grating inscription plane of the poled microreosonator. The focal spot is raster-scanned across the plane and the generated SH signal is collected vertically, thereby obtaining the *χ*^(2)^ grating images.

## Supplementary information

Supplementary Information

Transparent Peer Review file

## Data Availability

The data that support the plots within this paper are available at 10.6084/m9.figshare.28595867.v3.

## Code availability

The code used to produce the results of this paper are available at 10.6084/m9.figshare.28595867.v3.
