# Supplementary material for: Self-organized spatiotemporal quasi-phase-matching in microresonators
Source: Nat Commun. 2025 May 1;16:4083. doi: 10.1038/s41467-025-59215-1 (PMC12045971; doi:10.1038/s41467-025-59215-1)
Supplement: Supplementary file 1 — Supplementary Information [file 41467_2025_59215_MOESM1_ESM.pdf]

Supplementary Information for: Self-organized spatiotemporal quasi-phase-matching in microresonators

Ji Zhou[1,*], Jianqi Hu[1,2,*,†], Marco Clementi[1,3,*], Ozan Yakar[1], Edgars Nitiss[1], Anton Stroganov[4] & Camille-Sophie Brès[1‡]

[1]*Photonic Systems Laboratory (PHOSL), STI-IEM, École Polytechnique Fédérale de Lausanne, 1015 Lausanne, Switzerland.*

[2]*Present address: Department of Electrical and Electronic Engineering, The University of Hong Kong, Hong Kong, China.*

[3]*Present address: Dipartimento di Fisica "A. Volta", Università di Pavia, Via A. Bassi 6, 27100 Pavia, Italy.*

[4]*LIGENTEC SA, EPFL Innovation Park, 1024 Ecublens, Switzerland.*

[*] *These authors contributed equally to the work.*

[†] *Email: jianqi@hku.hk*

[‡] *Email: camille.bres@epfl.ch*

Contents

Supplementary Note I: Theoretical model 2

Supplementary Note II: SHG perturbed by a neighboring SH mode 10

Supplementary Note III: Power dependence 12

Supplementary Note IV: Additional experimental results 14

Supplementary Note I: Theoretical model

In this section, we provide the theoretical framework for describing the temporal dynamics of photoinduced SHG in Si_3N_4 microresonators. As depicted in Fig. 1c of the main text, this phenomenon arises from the interplay between CPE and EFISHG effect, which are modeled separately in the following.

Nonlinear $\chi^{(3)}$ processes in the presence of a DC electric field. The $\chi^{(3)}$ related nonlinear polarizability P_{NL} in the presence of both a static DC field E_{dc} and an optical field E_ω can be expressed as[1]:

$$\begin{aligned} P_{\text{NL}} &= \epsilon_0 \chi^{(3)} \left(E_{\text{dc}} + E e^{-i\omega t} + E^* e^{i\omega t} \right)^3 \\ &= P_{\text{Electrostatic}} + P_{\text{Kerr}} + P_{\text{DC}-\text{Kerr}} + P_{\text{EFISHG}} + P_{\text{THG}}, \end{aligned} \tag{S1}$$

with

$$\begin{cases} P_{\text{Electrostatic}} = \epsilon_0 \chi^{(3)} \left[E_{\text{dc}}^3 + 6|E|^2 E_{\text{dc}} \right] \\ P_{\text{Kerr}} = \epsilon_0 \chi^{(3)} \left[3|E|^2 E e^{-i\omega t} + 3|E|^2 E^* e^{i\omega t} \right] \\ P_{\text{DC- Kerr}} = \epsilon_0 \chi^{(3)} \left[3E_{\text{dc}}^2 E e^{-i\omega t} + 3E_{\text{dc}}^2 E^* e^{i\omega t} \right] \\ P_{\text{EFISHG}} = \epsilon_0 \chi^{(3)} \left[3E_{\text{dc}} E^2 e^{-i2\omega t} + 3E_{\text{dc}} \left(E^* \right)^2 e^{i2\omega t} \right] \\ P_{\text{THG}} = \epsilon_0 \chi^{(3)} \left[E^3 e^{-i3\omega t} + \left(E^* \right)^3 e^{i3\omega t} \right], \end{cases} \tag{S2}$$

where ϵ_0 is the vacuum permittivity. The polarizability comprises several components, i.e. $P_{\text{Electrostatic}}$, P_{Kerr}, $P_{\text{DC}-\text{Kerr}}$, P_{EFISHG}, and P_{THG}, each corresponding to the response of the material at different frequencies. We will foucs our attention on the EFISHG effect, that results in a polarizability oscillating at the SH frequency. Here, in analogy to the regular SHG process, an electric field induced effective $\chi^{(2)}$ can be written as $\chi^{(2)}_{\text{eff}} = 3\chi^{(3)} E_{\text{dc}}$. In the rest of our treatment to the efficient SHG in Si_3N_4 microresonators, we consider only the relevant Kerr, DC-Kerr, and EFISHG effects.

Photoinduced electric field from coherent photogalvanic effect. In this part, the analytical equation for the CPE is presented. The first-order CPE involves the generation of an anisotropic coherent current (j_{ph}) from the multiphoton absorption interference between the fundamental pump and its second harmonic (SH)[2–4]. In cylindrical coordinates (r, ϕ, z), the generated photogalvanic

current j_{ph} can be written as:

$$j_{\text{ph}}(r,\phi,z) = \beta\left(I_{\text{pump}}, I_{\text{SH}}\right)\left[E^*_{\text{pump}}(r,\phi,z)\right]^2 E_{\text{SH}}(r,\phi,z)e^{i\Delta k\phi R - i\psi_{\text{ph}}} + c.c., \tag{S3}$$

where $\beta\left(I_{\text{pump}}, I_{\text{SH}}\right) = \sum_{a,b}\beta_{ab}I^a_{\text{pump}}I^b_{\text{SH}}(a,b = 0,1,\ldots)$ is the generic photogalvanic coefficient with $I_{\text{pump, SH}}$ the intensities of light, $E_{\text{pump, SH}}$ are the amplitudes of optical electric fields, $\Delta k = k_{\text{SH}} - 2k_{\text{pump}}$ represents the wave vector difference, ϕ denotes the azimuthal angle along the circumference, R is the resonator radius, $\psi_{\text{ph}} = \pi$ represents the CPE interaction phase[4]. $c.c.$ stands for the complex conjugate.

In the absence of externally applied fields, the photogalvanic current will eventually be compensated by a drift current, which according to Ohm's law takes the form $j_{\text{drift}} = \sigma E_{\text{dc}}$ (note that j_{ph} and j_{drift} have opposite directions). A quasi-static electric field E_{dc} is therefore established inside the material as a consequence of the CPE, which facilitates the EFISHG process. When ignoring charge diffusion[5,6], the dynamics of the amplitude of the photoinduced electric field E_{dc} can be expressed as[4]:

$$\frac{\partial E_{\text{dc}}(r,\phi,z)}{\partial t} = \frac{j_{\text{ph}}(r,\phi,z)}{\epsilon_{\text{dc}}} - \frac{\sigma\left(I_{\text{pump}}, I_{\text{SH}}\right)E_{\text{dc}}(r,\phi,z)}{\epsilon_{\text{dc}}}, \tag{S4}$$

where $\epsilon_{\text{dc}} = \epsilon_0\epsilon_r$ represents the permittivity of the medium with ϵ_r the relative permittivity of the material at low frequency. $\sigma\left(I_{\text{pump}}, I_{\text{SH}}\right) = \sum_{a,b}\sigma_{ab}I^a_{\text{pump}}I^b_{\text{SH}}$ is the intensities dependent photoconductivity.

Coupled mode equations for resonant SHG. For convenience, in the following we use the subscripts $\{p,s,i\}$ to denote the intracavity optical pump field, intracavity optical SH field and photoinduced electric field, respectively.

Based on the combination of CPE and EFISHG effects discussed above, the SHG in Si_3N_4

microresonators can be described by the following coupled mode theory[7,8]:

$$\begin{cases} \dfrac{\partial A_p(t)}{\partial t} = -\left(\dfrac{\kappa_p}{2} + i\delta_p\right) A_p + \sqrt{\kappa_p \eta_p P_{\text{in}}} \\ \qquad\qquad + i\left(\gamma_{pppp}\left|A_p\right|^2 + 2\gamma_{psps}\left|A_s\right|^2 + 2\gamma_{pipi}\left|A_i\right|^2\right) A_p + i\left(2\gamma_{pspi}A_i^*\right) A_s A_p^* \\ \dfrac{\partial A_s(t)}{\partial t} = -\left(\dfrac{\kappa_s}{2} + i\delta_s\right) A_s \\ \qquad\qquad + i\left(\gamma_{ssss}\left|A_s\right|^2 + 2\gamma_{spsp}\left|A_p\right|^2 + 2\gamma_{sisi}\left|A_i\right|^2\right) A_s + i\left(\gamma_{spip}A_i\right) A_p^2 \\ \dfrac{\partial A_i(t)}{\partial t} = \beta'\left(A_p^*\right)^2 A_s e^{-i\psi_{\text{ph}}} - \dfrac{A_i}{\tau}, \end{cases} \tag{S5}$$

where $|A_{p,s}|^2 = \int_V dv\epsilon_{p,s}\left|E_{p,s}\right|^2$ represent the modal energy of optical pump and SH fields, $|A_i|^2 = \frac{1}{2}\int_V dv\epsilon_i\left|E_i\right|^2$ is also the energy of the photoinduced electric field considering its spatial distribution despite not being a resonant mode (see Eq. (S3, S4)). $\epsilon_{p,s} = \epsilon_0 n_{p,s}^2$ with $n_{p(s)}$ the refractive indices of Si_3N_4 waveguide for pump and SH.

For the other parameters in Eq. (S5), κ_p and κ_s are the total loss rates of optical pump and SH fields, respectively. $\delta_p = \omega_p - \omega_{\text{pump}}$ and $\delta_s = \omega_s - 2\omega_{\text{pump}}$ are the detunings for pump resonance (ω_p) and the SH resonance (ω_s). $\eta_p = \kappa_{\text{ex},p}/\kappa_p$ represents the coupling efficiency of the pump field with $\kappa_{\text{ex},p}$ the output coupling rate. $\gamma_{jklm}\left(E_j, E_k, E_l, E_m = E_p, E_s, E_i^*\right)$ denote the third-order nonlinear parameters defined in a similar manner as in Refs. 7, 8. $\tau = \epsilon_i/\sigma$ is the lifetime of photoinduced electric field or inscribed nonlinear grating. Additionally, $\beta' = \frac{\xi}{\epsilon_p\sqrt{2\epsilon_s\epsilon_i}}\beta$ with ξ the nonlinear overlap defined as:

$$\xi = \sqrt{\frac{\int_V dv\left|\left(E_p^*\right)^2 E_s\right|^2}{\left(\int_V dv\left|E_p\right|^2\right)^2 \int_V dv\left|E_s\right|^2}}. \tag{S6}$$

In Eq. (S5), the nonlinear terms in the first two equations describe self-phase modulation (SPM), cross-phase modulation (XPM), DC-Kerr and EFISHG effects, respectively. Derived from Eq. (S4), the third equation describes the dynamics of the photoinduced electric field. It is worth noting that in this context, QPM is inherently satisfied among these three fields, the phase mismatch terms can be reduced by variable substitution.

The Si_3N_4 microresonantor utilized in this work is identical to the one (with FSR of 146 GHz) in ref. 9, and we use the same material parameters from Refs. 4, 10. Table S1 lists the basic information of the Si_3N_4 microresonator as well as the intensity-dependent photogalvanic coefficients and photoconductivity coefficients used in the simulation.

Table S1: Parameters used in simulations.

Parameters	Value
microresonantor cross-section, $h \times w$	$1.7 \times 0.5\ \mu\text{m}^2$
ring radius, R	$158\ \mu\text{m}$
third-order susceptibility[8], $\chi^{(3)}_{spip}$	$3.39 \times 10^{-21}\ \text{m}^2/\text{V}^2$
CPE interaction phase, ψ_{ph}	π rad
photoconductivity coefficients[4], σ_{mn}	$\begin{cases} \sigma_{00} \approx 5.12 \times 10^{-17}\ \text{S}\cdot\text{m}^{-1}\ {}^{*} \\ \sigma_{02} = 1.19 \times 10^{-33}\ \text{S}\cdot\text{m}^3\cdot\text{W}^{-2} \\ \sigma_{21} = 1.36 \times 10^{-48}\ \text{S}\cdot\text{m}^5\cdot\text{W}^{-3} \end{cases}$
photogalvanic coefficients[4], β_{mn}	$\begin{cases} \beta_{01} = 8.22 \times 10^{-38}\ \text{m}^3\cdot\text{V}^{-4} \\ \beta_{20} = 6.17 \times 10^{-53}\ \text{m}^5\cdot\text{V}^{-4}\cdot\text{W}^{-1} \end{cases}$

* $\sigma_{00} = \epsilon_i/\tau_{00}$ with τ_{00} being approximately 15 days[10].

Traveling grating and AOP condition. An intriguing property of the system descried by Eq. (S5) is that it can sustain a nonstationary steady state, which implies that the photoinduced electric field is traveling at the equilibrium. We can analyze the system by writing the Jacobian matrix of Eq. (S5) as:

$$\boldsymbol{M} = \begin{pmatrix} -\left(\frac{\kappa_p}{2} + i\delta'_p\right) & 0 & 0 & 0 & 0 & 0 \\ i2\gamma_{spip}A_pA_i & -\left(\frac{\kappa_s}{2} + i\delta'_s\right) & i\gamma_{spip}A_p^2 & 0 & 0 & 0 \\ 0 & \beta'(A_p^*)^2e^{-i\psi_{\text{ph}}} & -\frac{1}{\tau} & 2\beta' A_p^* A_s e^{-i\psi_{\text{ph}}} & 0 & 0 \\ 0 & 0 & 0 & -\left(\frac{\kappa_p}{2} - i\delta'_p\right) & 0 & 0 \\ 0 & 0 & 0 & -i2\gamma_{spip}A_p^*A_i^* & -\left(\frac{\kappa_s}{2} - i\delta'_s\right) & -i\gamma_{spip}\left(A_p^*\right)^2 \\ 2\beta' A_p A_s^* e^{i\psi_{\text{ph}}} & 0 & 0 & 0 & \beta' A_p^2 e^{i\psi_{\text{ph}}} & -\frac{1}{\tau} \end{pmatrix}. \quad \text{(S7)}$$

Here we group all the nonlinear phase terms in Eq. (S5) to the effective detunings (δ_p', δ_s'), which can also include the thermo-optic effects, though not specifically described in Eq. (S5).

The characteristic equation for the eigenvalues of $\boldsymbol{M}$ is:

$$\begin{aligned}&\left[\left(\tfrac{\kappa_p}{2}+i\delta_p'\right)+\lambda\right]\left\{\left[\left(\tfrac{\kappa_s}{2}+i\delta_s'\right)+\lambda\right]\left(\tfrac{1}{\tau}+\lambda\right)+i\varUpsilon\left|A_p\right|^4\right\}\\ &\cdot\left[\left(\tfrac{\kappa_p}{2}-i\delta_p'\right)+\lambda\right]\left\{\left[\left(\tfrac{\kappa_s}{2}-i\delta_s'\right)+\lambda\right]\left(\tfrac{1}{\tau}+\lambda\right)-i\varUpsilon\left|A_p\right|^4\right\}=0,\end{aligned} \tag{S8}$$

where $\varUpsilon=\gamma_{spip}\beta'$.

Under the assumptions of $\left|i\varUpsilon A_p^4\right|\ll\left|\frac{\kappa_s}{2}+i\delta_s'\right|$ and $\frac{1}{\tau}\ll\left|\frac{\kappa_s}{2}+i\delta_s'\right|$ (the photoinduced electric field exhibits much slower growth or decay rates compared to the loss rates and detunings of optical fields), the first three eigenvalues from the first two terms of Eq. (S8) are given by:

$$\begin{cases}\lambda_1=-\left(\frac{\kappa_p}{2}+i\delta_p'\right)\\ \lambda_2\approx-\left(\frac{\kappa_s}{2}+i\delta_s'\right)\\ \lambda_3\approx-\frac{1}{\tau}-\frac{i\varUpsilon|A_p|^4}{\kappa_s/2+i\delta_s'}=-\frac{\varUpsilon|A_p|^4\delta_s'}{\kappa_s^2/4+(\delta_s')^2}-\frac{1}{\tau}-i\frac{\varUpsilon|A_p|^4\kappa_s/2}{\kappa_s^2/4+(\delta_s')^2},\end{cases} \tag{S9}$$

The nonvanishing imaginary part of λ_3 gives the oscillation frequency (Ω) of the photoinduced field:

$$\operatorname{Im}\left\{\lambda_3\right\}\approx-\frac{\varUpsilon\left|A_p\right|^4\kappa_s/2}{\kappa_s^2/4+\left(\delta_s'\right)^2}=\Omega<0. \tag{S10}$$

The additional relation between τ and Ω is given by the periodic solutions at steady state. The existence of non-stationary steady states is not theoretically proved here but consistently observed experimentally, we hence assume the form of the solution of Eq. (S5) at equilibrium is $A_p\rightarrow A_p$, $A_s\rightarrow A_se^{i\Omega t}$, $A_i\rightarrow A_ie^{i\Omega t}$, from Eq. (S5) we obtain:

$$\begin{cases}A_p=\frac{\sqrt{\kappa_p\eta_pP_{\text{in}}}}{\left(\frac{\kappa_p}{2}+i\delta_p'\right)}\\ i\Omega A_s=-\left(\frac{\kappa_s}{2}+i\delta_s'\right)A_s+i\left(\gamma_{spip}A_i\right)A_p^2\\ i\Omega A_i=\beta'\left(A_p^*\right)^2A_se^{-i\psi_{\text{ph}}}-\frac{A_i}{\tau}.\end{cases} \tag{S11}$$

This leads to

$$\begin{cases} \frac{\kappa_s}{2\tau} - (\delta_s' + \Omega)\,\Omega = 0 \\ \frac{\delta_s' + \Omega}{\tau} + \Omega\frac{\kappa_s}{2} = -\Upsilon\,|A_p|^4\,, \end{cases} \tag{S12}$$

In the limit where $|\delta_s'| \gg |\Omega|$, we can derive

$$\frac{1}{\tau} \approx -\frac{\Upsilon\,|A_p|^4\,\delta_s'}{\kappa_s^2/4 + (\delta_s')^2} = \frac{2\delta_s'}{\kappa_s}\Omega. \tag{S13}$$

The relation $\Omega \approx \frac{\kappa_s}{2\tau\delta_s'}$ in Eq. (S13) is used to fit the simulation results, as illustrated in Fig. 3 in the main text, and Fig. S2(b) as well as Fig. S4(a). More importantly, we can see that the condition for efficient SHG is $\delta_s' < 0$ as τ and κ_s are positive, while Ω remains negative.

In addition, from Eq. (S9) and Eq. (S13), we notice that $\mathrm{Re}\{\lambda_3\} \approx 0$ holds for the ansatz used in Eq. (S11), explaining the constant-power steady states reported in this work. Experimentally, we also observe other non-stationary states with periodic oscillations in SH power, likely due to the intricate CPE or the thermal effect. We leave the detailed study for future work.

Pump threshold and conversion efficiency. Different from conventional threshold-less cavity-enhanced SHG[11], the photoinduced SHG typically exhibits a threshold[8,9,12]. This threshold behavior can be attributed, in part, to the requirement for the inscribed $\chi^{(2)}$ grating from CPE to surpass its erasure mediated by conductivity. Here we simulate the SHG threshold using the dark conductivity σ_{00} of the material, while omitting for simplicity the potential contributions of the photoconductivity from the pump (e.g., σ_{40}).

When pumped above the threshold power, the system loses its stability and evolves from the state without SHG ($A_s = A_i = 0$) to the state that generates SH. Mathematically, it writes:

$$\mathrm{Re}\,\{\lambda_3\} \approx -\frac{\Upsilon\,|A_p|^4\,\delta_s'}{\kappa_s^2/4 + (\delta_s')^2} - \frac{1}{\tau} \geqslant 0. \tag{S14}$$

As the parameters $\Upsilon = \gamma_{spip}\beta'$ and $\tau = \epsilon_i/\sigma$ depend on the light intensities (see Table S1),

with the relation $A_p = \frac{\sqrt{\kappa_p \eta_p P_{\rm in}}}{\kappa_p/2 + i\delta'_p}$ the pump threshold power in the bus waveguide can be derived as:

$$P_{\rm th}(\delta'_p, \delta'_s) = \sqrt[4]{\frac{\sigma_{00}\left(\kappa_p^2/4 + (\delta'_p)^2\right)^4 \left(\kappa_s^2/4 + (\delta'_s)^2\right)}{\mathcal{L}\delta'_s}}, \tag{S15}$$

where $\mathcal{L} = \frac{\gamma_{spip}\xi\kappa_{{\rm ex},p}^4 \beta_{20} e^{-i\psi_{\rm ph}}}{\epsilon_p}\sqrt{\frac{\epsilon_i}{2\epsilon_s}}\left(\frac{D_{1,p}}{2\pi A_{{\rm eff},p}}\right)^2$ with $D_{1,p}/2\pi$ and $A_{{\rm eff},p}$ representing the FSR and effective mode area of the pump field, respectively. Note that the pump threshold is minimized for $(\delta'_p, \delta'_s) = (0, -\frac{\kappa_s}{2})$, which represents the optimal operating point. The threshold pump power discussed here is considered in the context of the absence of SH light. It can be reduced by seeding coherent SH light[4,6] or poling the microresonator beforehand[13,14].

The simulated threshold power as a function of detunings is graphically illustrated in Fig. S1(a). The threshold power exhibits a stronger dependence on pump detuning δ'_p than SH detuning δ'_s, and it could vary approximately an order of magnitude when the pump detuning is changed over a linewidth at pump. Based on the simulation, we estimate that the detuning-dependent threshold power ranges from 1×10^{-3} to 2×10^{-2} W, which roughly aligns with the previously observed

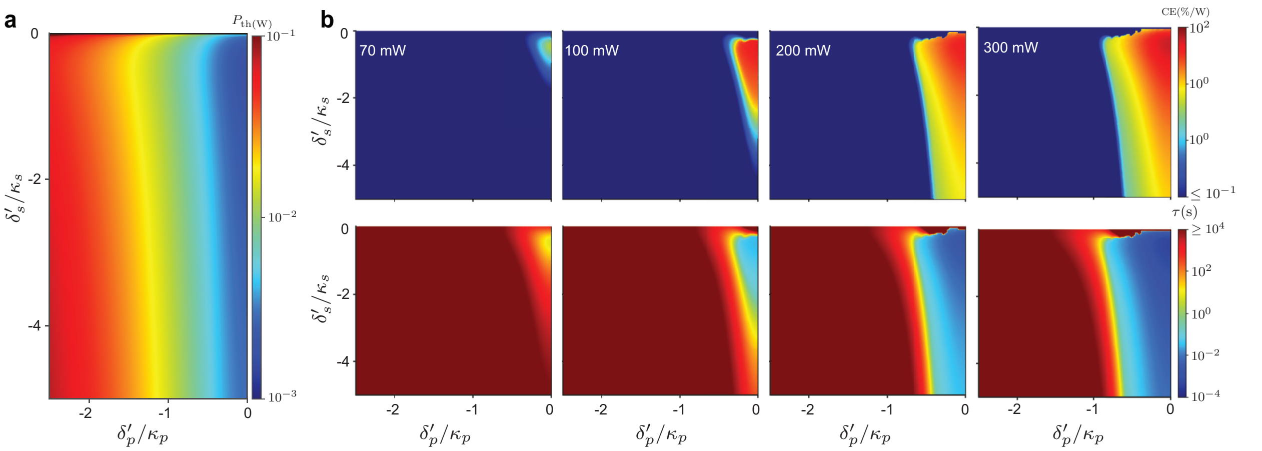

Figure S1: Simulation of threshold behavior of photo-induced SHG. a, Simulated threshold power $P_{\rm th}$ as a function of pump and SH detunings. **b**, Conversion efficiency (CE) map (top) and grating lifetime τ (bottom) as functions of detunings under different pump power. In simulation, the $\rm TE_{00}$ mode for pump around $1542.7\ \rm nm$ and $\rm TE_{30}$ mode for SH and are considered[9], with simulation parameters $Q_p\,(Q_s) = 0.73\ (1.30) \times 10^6, \kappa_p\,(\kappa_s)/2\pi = 270\ (300)\ \rm MHz, \eta_p\,(\eta_s) = 0.5\ (0.5)$ used.

value of approximately 2.5×10^{-2} W[9]. Table S2 summarizes the comparison of threshold power observed in different studies. In microresonators, the threshold pump power P_{th} scales inversely with the finesse $\mathcal{F} = D_{1,p}/\kappa_p$ assuming optimal detuning conditions, as derived from Eq. (S15).

The upper panel in Fig. S1(b) shows the simulated conversion efficiency CE $= P_{s,\text{out}}/P_{p,\text{in}}^2$ with $P_{s,\text{out}}$ and $P_{p,\text{ in}}$ being the generated SH and the pump power in the bus waveguide, respectively. The lifetime τ as a function of detunings is also plotted in the bottom panel. Similar to the saturation behavior of CE in conventional SHG[11], the CE in our system is clamped at approximately $60\%/\text{W}$, given the simulation parameters listed in the caption. Note that in our simulations, the back conversion from SH to pump leading to pump depletion is ignored. The saturation behavior of CE is attributed to the coherent photogalvanic effect.

Qualitatively, as the intracavity optical power increases, both the photogalvanic coefficient β (related to the 'gain' of the photoinduced electric field A_i) and conductivity σ ('loss' of A_i) will increase. Above a certain level, further increase in the pump power will contribute more to the photoconductivity than to the photogalvanic coefficient and eventually clamp the CE.

Table S2: Comparison of the threshold power for photoinduced SHG in different works.

References	Pump finesse, $\mathcal{F}$	P_{th} (W)
microring[9]	545	$< 2.5 \times 10^{-2}$
microring[12]	6472	$< 4 \times 10^{-3}$
microring[8]	5000	2.3×10^{-3}
single-pass waveguide[15]	–	< 9.84
single-pass waveguide[10]	–	< 38
single-pass waveguide[16]	–	< 60

* Additional comparisons are available in Ref. 8.

Supplementary Note II: SHG perturbed by a neighboring SH mode

Experimentally, there are instances where the frequency offset is larger than zero as shown in the Fig. 2b in the main text, which is not supported by the theory we establish above (Eq. (S10)). A possible explanation is the participation of another neighboring SH mode in the SHG process. Considering two following facts: i) from the VNA response maps in Fig. 3a and c of the main text and also Fig. S2(b), it is evident that during SHG, the pump is thermally locked to its nearest resonance (see the nearly horizontal pump branch), while the generated SH is not (the tilted SH branch)[9]. This implies that the SH detuning could shift a lot by several linewidths. ii) In a multimode Si_3N_4 waveguide, a multitude of transverse modes at SH band show distinct thermo-optic coefficients, and it is possible that sometimes a neighboring SH mode becomes more resonant during the frequency tuning at pump. These reasonable assumptions can lead to simultaneous SHG for multiple SH modes, which may explain the phenomenon of $\Omega > 0$ we observe in the experiment.

Here we consider the scenario where the electric field is composed of two components ($A_{i,q}$). In this case, Eq. (S5) can be extended as:

$$\begin{cases} \frac{\partial A_p}{\partial t} = -\left(\frac{\kappa_p}{2} + i\delta'_p\right) A_p + \sqrt{\kappa_p \eta_p P_{\text{in}}} \\ \frac{\partial A_s}{\partial t} = -\left(\frac{\kappa_s}{2} + i\delta'_s\right) A_s + i\left(\gamma_{spip} A_i\right) A_p^2 \\ \frac{\partial A_i}{\partial t} = \beta'_s \left(A_p^*\right)^2 A_s e^{-i\psi_{\text{ph}}} - \frac{A_i}{\tau_i} + i g^* A_q \\ \frac{\partial A_w}{\partial t} = -\left(\frac{\kappa_w}{2} + i\delta'_w\right) A_w + i\left(\gamma_{wpqp} A_q\right) A_p^2 e^{i\psi} \\ \frac{\partial A_q}{\partial t} = \beta'_w \left(A_p^*\right)^2 A_w e^{-i\psi_{\text{ph}}} - \frac{A_q}{\tau_q} + i g A_i, \end{cases} \tag{S16}$$

where $\{A_p, A_s, A_i\}$ and $\{A_p, A_w, A_q\}$ are QPM mode pairs, g is the linear coupling coefficient related to the charge diffusion, ψ is the spatial phase difference between the two electric field components.

For simplicity, we ignore the linear coupling between photoinduced electric fields ($g = 0$). Generally, one SH mode (A_s) dominates the SHG process and the other one (A_w) can be considered

as a perturbation. To simplify the simulation, we assume the ratio between the two electric field strengths is fixed ($\alpha = A_q/A_i \ll 1$), such that Eq. (S16) can be reduced to four equations by adding the two photoinduced electric field equations:

$$\begin{cases} \dfrac{\partial A_p}{\partial t} = -\left(\dfrac{\kappa_p}{2} + i\delta_p'\right) A_p + \sqrt{\kappa_p \eta_p P_{\text{in}}} \\ \dfrac{\partial A_s}{\partial t} = -\left(\dfrac{\kappa_s}{2} + i\delta_s'\right) A_s + i\left(\gamma_{spip} A_i\right) A_p^2 \\ \dfrac{\partial A_w}{\partial t} = -\left(\dfrac{\kappa_w}{2} + i\delta_w'\right) A_w + i\left[\gamma_{wpqp}\left(\alpha A_i\right)\right] A_p^2 e^{i\psi} \\ \dfrac{\partial A_i}{\partial t} \approx \beta_s' \left(A_p^*\right)^2 A_s e^{-i\psi_{\text{ph}}} + \beta_w' \left(A_p^*\right)^2 A_w e^{-i\psi_{\text{ph}}} - \dfrac{A_i}{\tau}, \end{cases} \tag{S17}$$

where $1/\tau = 1/\tau_i + \alpha/\tau_q$ and we set $\alpha = 0.1$ in our simulation.

Similar to the derivation of Eq. (S9), we can obtain the expression of the eigenvalue related to the evolution of photoinduced electric field at the steady state:

$$\lambda_4 \approx -\frac{1}{\tau} - \frac{i\alpha \Upsilon_w \left|A_p\right|^4 e^{i\psi}}{\kappa_w/2 + i\delta_w'} - \frac{i\Upsilon_s \left|A_p\right|^4}{\kappa_s/2 + i\delta_s'}. \tag{S18}$$

We notice that the eigenvalue in Eq. (S18) is now perturbed by the adjacent SH mode, as compared to Eq. (S9). In the simulation shown in Fig. S2, we assume ψ takes the value of $\pi/2$ and calculate the frequency offset by $\Omega = \mathrm{Im}\left\{\lambda_4\right\}$ for the fitting.

Figure S2 shows the simulated zero-crossing behavior of the frequency offset. The presence of two neighboring different order modes at SH band is considered. When tuning the pump wavelength, the SH mode λ_{s2} will start to effectively perturb the existing SH generated from the mode λ_{s1} at a certain pump wavelength, resulting in $\Omega > 0$. In addition, such a zero-crossing behavior also exhibits pump power dependence (see Supplementary Note III for more details).

Further, we anticipate that if the detuning of the neighboring SH mode becomes small enough, competition between the two modes may occur, until reaching a stable state where the adjacent mode takes over the SHG. Such a phenomenon has already been observed previously (Fig. S8 in ref. 9) and further investigation is left for future work.

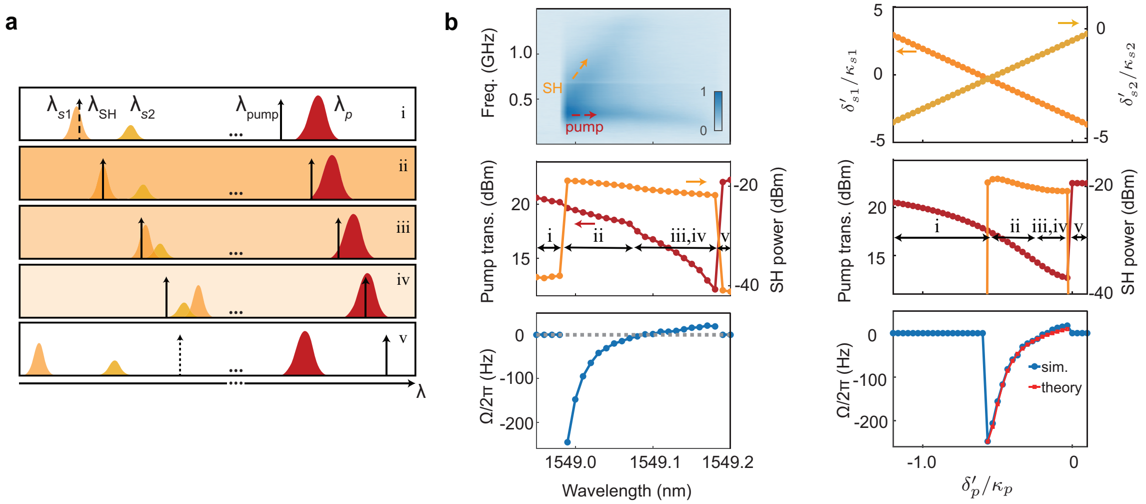

Figure S2: Dynamics of AOP-enabled SHG in the presence of a neighboring SH mode. a, Schematic illustration of the pump and SH resonance distributions with respect to the pump and SH signals, respectively. **b**, Experimental (left) and simulation (right) results. δ'_{s1} and δ'_{s2} denote the effective detunings of the main and the perturbing SH modes, respectively. In the simulation, the frequency offset is also fitted by the theoretical prediction $\Omega = \mathrm{Im}\{\lambda_4\}$.

Apart from the perturbation from a neighboring SH mode considered above, sum-frequency generation[17], third-harmonic generation[18] and other nonlinear processes may also lead to the zero-crossing behavior through nonlinear mode interaction.

Supplementary Note III: Power dependence

In this section, we show the power-dependent behaviours of the photo-induced SHG. Figure S3 shows the measurement results under different pump power levels for the resonance near 1543.0 nm, which belongs to the leading case. It can be seen that higher pump power here corresponds to less efficient SHG, as shown in the left upper and middle panels in Fig. S3(a). Note that at the same pump wavelength, the generated SH power decreases with increasing pump power. This is because higher pump power shifts the SH resonance farther away from the generated SH frequency via the thermal and Kerr effects. In addition, given that the photoconductivity depends more on the intensity of SH than that of pump[4], the grating lifetime would decrease with the generated SH

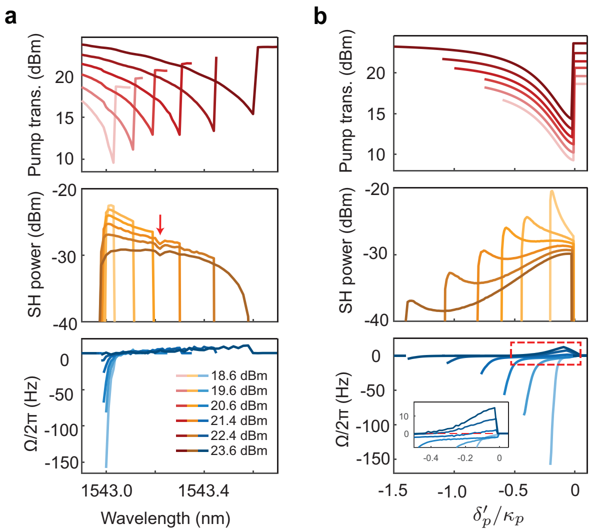

Figure S3: SHG characteristics at the resonance near 1543.0 nm under different pump power. **a**, Top: Measured pump transmission; Middle: Measured generated SH power; Bottom: Measured frequency offsets. In the experiments, the generated SH power decreases when increasing the pump power or tuning the pump into the resonance. Above a certain pump power level, discrete changes in the generated SH power (denoted by the red arrow) and the zero-crossing of the frequency offset are observed. **b**, Top: Simulated pump transmission; Middle: Simulated generated SH power; Bottom: Simulated frequency offsets. In the simulation, the mode perturbation is introduced to the cases with pump power levels of 21.4, 22.4, and 23.6 dBm to reproduce the zero-crossing behaviors, as shown in the inset.

power, i.e., $\tau \propto \left(\sigma_{00} + \sigma_{02} I_{\mathrm{SH}}^2 + \sigma_{21} I_{\mathrm{pump}}^2 I_{\mathrm{SH}}\right)^{-1}$. The combined effects explain the experimental observations that lower pump power corresponds to a higher frequency shift since $\Omega \propto \frac{1}{\delta'_s \tau}$.

Experimentally, the zero crossing behaviour of the measured frequency offset becomes increasingly evident at high pump power. As discussed in Supplementary Note II, we attribute the zero crossing to the perturbation of a neighboring SH mode, which are present in Fig. 2b of the main text as well. The small drop in the generated SH power trace, marked by the red arrow in Fig. S3(a), may be a signature of the change of the rotating direction of the grating when the frequency offset Ω changes the sign. In Fig. S3(b), we perform simulations to reproduce the experimental results. In the simulation, perturbations from a neighboring lossy resonance with a large linewidth are taken into account for the cases using 21.4, 22.4, and 23.6 dBm pump power. It can

be seen that the frequency offset zero-crossing behaviors up to few Hz are qualitatively reproduced in the simulation.

Supplementary Note IV: Additional experimental results

Apart from the experimental results that correspond to the leading and trailing cases in the main text, there are also other scenarios with one of the examples shown in Fig. S4. Fig. S4 illustrates the AOP dynamics for the pump wavelength around 1552.2 nm, where the position of the maximum absolute value of the frequency offset does not align with either the leading or the trailing edge of the generated SH power trace. This behavior, however, does not contradict the presented theory (Eq. (S13)), as the frequency offset does not strictly follow an inversely proportional relation to

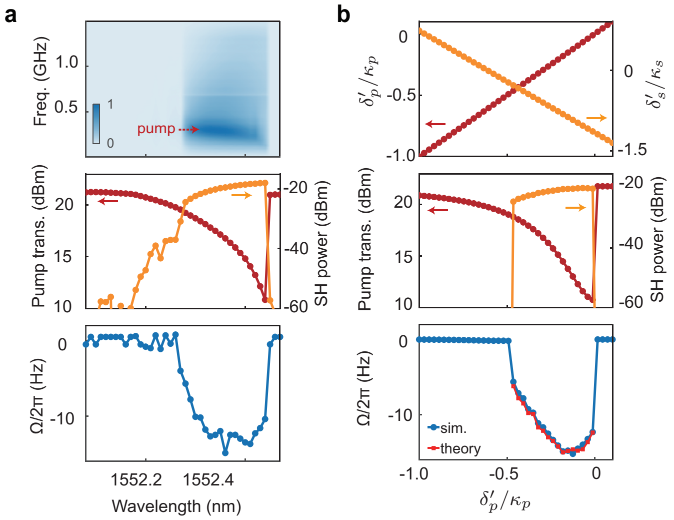

Figure S4: Additional measurement and simulation results of photoindcued SHG in the Si_3N_4 microresonator. For the AOP around 1552.2 nm, the maximum absolute value of the frequency offset ($\Omega_{\max}$) occurs at the middle of the SH power trace. **a**, Top: VNA response map; Middle: Measured pump transmission and generated SH power; Bottom: Measured frequency offset ($\Omega/2\pi$). The experimental non-sharp leading edge of SH power trace may be attributed to SHG from a preceding SH mode or less efficient SHG from a not fully erased $\chi^{(2)}$ grating. **b**, Top: the pump and SH effective detunings setting in the simulation; Middle: Simulated pump transmission and generated SH power; Bottom: Simulated frequency offset and the theoretical prediction based on Eq. (S13).

the SH detuning. This is because the grating lifetime τ, which depends on power, is also related to the detuning. In this specific case, for which the participation SH mode is identified by TPM imaging as TE_{40}, we expect that τ becomes significant given that this resonance corresponds to the highest SH power[9] and hence start significantly influence the frequency offset.

References

1. Friedman, A., Nejadriahi, H., Sharma, R. & Fainman, Y. Demonstration of the DC-Kerr effect in silicon-rich nitride. *Opt. Lett.* **46**, 4236–4239 (2021). URL https://opg.optica.org/ol/abstract.cfm?URI=ol-46-17-4236.

2. Anderson, D. Z., Mizrahi, V. & Sipe, J. E. Model for second-harmonic generation in glass optical fibers based on asymmetric photoelectron emission from defect sites. *Opt. Lett.* **16**, 796–798 (1991). URL https://opg.optica.org/ol/abstract.cfm?URI=ol-16-11-796.

3. Dianov, E. M. & Starodubov, D. S. Photoinduced generation of the second harmonic in centrosymmetric media. *Quantum Electronics* **25**, 395 (1995). URL https://dx.doi.org/10.1070/QE1995v025n05ABEH000371.

4. Yakar, O., Nitiss, E., Hu, J. & Brès, C.-S. Generalized coherent photogalvanic effect in coherently seeded waveguides. *Laser Photonics Rev.* **16**, 2200294 (2022). URL https://onlinelibrary.wiley.com/doi/abs/10.1002/lpor.202200294.

5. Zabelich, B., Nitiss, E., Stroganov, A. & Bres, C. S. Linear electro-optic effect in silicon nitride waveguides enabled by electric-field poling. *ACS Photonics* **9**, 3374–3383 (2022). URL https://www.ncbi.nlm.nih.gov/pubmed/36281331.

6. Yakar, O., Nitiss, E., Hu, J. & Brès, C.-S. Integrated backward second-harmonic generation through optically induced quasi-phase-matching. *Phys. Rev. Lett.* **131**, 143802 (2023). URL https://link.aps.org/doi/10.1103/PhysRevLett.131.143802.

7. Lin, Q., Johnson, T. J., Perahia, R., Michael, C. P. & Painter, O. J. A proposal for highly tunable optical parametric oscillation in silicon micro-resonators. *Opt. Express* **16**, 10596–10610 (2008). URL https://opg.optica.org/oe/abstract.cfm?URI=oe-16-14-10596.

8. Lu, X. & Srinivasan, K. Considering photoinduced second-harmonic generation as a dc Kerr optical parametric oscillation or amplification process. *Phys. Rev. Appl.* **16**, 014027 (2021). URL https://link.aps.org/doi/10.1103/PhysRevApplied.16.014027.

9. Nitiss, E., Hu, J., Stroganov, A. & Brès, C.-S. Optically reconfigurable quasi-phase-matching in silicon nitride microresonators. *Nat. Photonics* **16**, 134–141 (2022). URL https://doi.org/10.1038/s41566-021-00925-5.

10. Nitiss, E. *et al.* Formation rules and dynamics of photoinduced $\chi^{(2)}$ gratings in silicon nitride waveguides. *ACS Photonics* **7**, 147–153 (2020). URL https://doi.org/10.1021/acsphotonics.9b01301.

11. Guo, X., Zou, C.-L. & Tang, H. X. Second-harmonic generation in aluminum nitride microrings with 2500%/w conversion efficiency. *Optica* **3**, 1126–1131 (2016). URL https://opg.optica.org/optica/abstract.cfm?URI=optica-3-10-1126.

12. Lu, X., Moille, G., Rao, A., Westly, D. A. & Srinivasan, K. Efficient photoinduced second-harmonic generation in silicon nitride photonics. *Nat. Photonics* **15**, 131–136 (2020). URL https://doi.org/10.1038/s41566-020-00708-4.

13. Li, B. *et al.* High-coherence hybrid-integrated 780 nm source by self-injection-locked second-harmonic generation in a high-Q silicon-nitride resonator. *Optica* **10**, 1241–1244 (2023). URL https://opg.optica.org/optica/abstract.cfm?URI=optica-10-9-1241.

14. Clementi, M. *et al.* A chip-scale second-harmonic source via self-injection-locked all-optical poling. *Light Sci. Appl.* **12**, 296 (2023). URL https://doi.org/10.1038/s41377-023-01329-6.

15. Porcel, M. A. *et al.* Photo-induced second-order nonlinearity in stoichiometric silicon nitride waveguides. *Opt. Express* **25**, 33143–33159 (2017). URL https://opg.optica.org/oe/abstract.cfm?URI=oe-25-26-33143.

16. Billat, A. *et al.* Large second harmonic generation enhancement in Si_3N_4 waveguides by all-optically induced quasi-phase-matching. *Nat. Commun.* **8**, 1016 (2017). URL https://doi.org/10.1038/s41467-017-01110-5.

17. Hu, J. *et al.* Photo-induced cascaded harmonic and comb generation in silicon nitride microresonators. *Sci. Adv.* **8**, eadd8252 (2022). URL https://www.science.org/doi/abs/10.1126/sciadv.add8252.

18. Levy, J. S., Foster, M. A., Gaeta, A. L. & Lipson, M. Harmonic generation in silicon nitride ring resonators. *Opt. Express* **19**, 11415–11421 (2011). URL https://opg.optica.org/oe/abstract.cfm?URI=oe-19-12-11415.
